# Explanatory Note in Regard to Diagnosis of Blood Stains

**Published:** 1875-05

**Authors:** Jos. G. Richardson

**Affiliations:** Microscopist to the Pennsylvania Hospital


### EXPLANATORY NOTE IN REGARD TO THE DIAGNOSIS OF BLOOD STAINS.

By JOS. G. RICHARDSON, M.D.,

Microscopist to the Pennsylvania Hospital.

In an article by Dr. J. J. Woodward, in the last issue of this Journal, elicited by my paper on Blood Stains, published in the No. for July, 1874, it is stated that we can never "affirm truthfully on the strength of microscopical investigation that a given stain is positively composed of human blood." With this statement I fully agree, maintaining, however, that, whilst it is literally true, it is not the whole truth, because it often happens in practice, that *evidence other than microscopical*, narrows down the conditions of a case to the question: Is this stain human blood or that of an ox, pig, or sheep? The microscopist can then, in such cases, from fair specimens of blood spots, as ordinarily produced, affirm truthfully that "a given stain is positively composed of human blood," should it really be so, and this if doubted I can conclusively prove.

In respect to the *just* prominence which should be given to the circumstance that "the blood corpuscles of a few mammals approach so nearly in size to those of man as to render their distinction doubtful," a fact which I thus mention in my essay in this Journal for July, 1869, of which the paper of July, 1874, is a continuation (see also *Handbook of Med. Micros.*, p. 288), I think Dr. Woodward undervalues, in the first place, the prudence of our many medical brethren who possess microscopes without considering themselves experts; and, second, that he has overlooked in the calculation (which we both, perhaps equally, sought to make, of how to secure for humanity, by our researches, the greatest benefit with the least injury)

one important factor, viz., the shrewd-witted lawyer, to be found in every country town, who would infallibly see that not one syllable of the carefully worded statements in my paper supported any unqualified microscopist's claim to distinguish human from dog's or monkey's blood. Hence, trusting to this powerful element for the protection of the two or three innocent persons who might otherwise be endangered, I felt (honestly if mistakenly), whilst writing both my first paper and its continuation, that, should I more than indicate the animals which render our conclusions doubtful, my work might be utterly condemned as prejudicial to the interests of society, and myself perhaps compared (should I emphasize and reiterate the fact that science alone could not detect the falsehood of a criminal's story if he cunningly asserted that suspicious stains were made by the blood of a dog) to a toxicologist publishing a treatise, setting forth most faithfully the method by which poisoners may best destroy their victims with the least danger of detection in their crimes.* Be it remembered also, that in all cases a really innocent person, wrongly accused of murder, on the ground of blood stains upon his clothing, etc., actually produced from that "constant" (yet rarely slaughtered) "companion of man," the dog, or from a seal, or otter, needs no microscopist to prompt him into telling (and trying to confirm), *when first arrested*, the *true* origin of the suspicious blood spots.

These various considerations led me to publish my results in a guarded manner, but, now that all responsibility for harm has been removed, I am glad for the sake of the *few* who might draw erroneous inferences from my former papers, to say most emphatically, that I believe we cannot at present distinguish positively, in dried stains, between the blood corpuscles of man, and those of any mammal in which the disks measure on an average over $\frac{1}{4000}$ of an inch. Hence, therefore, until further discoveries are made, a microscopist's best efforts at revealing crime can only serve the cause of right and justice in those cases where the criminal's attorneys, in spite of being *forewarned* and consequently forearmed, fail to

* The gendarmerie of Valenciennes have just arrested a Dutchman whose profession is, to say the least of it, extraordinary. He is a dealer in all sorts of instruments employed by burglars and thieves. When arrested he had in his possession a large stock of pamphlets giving the fullest directions as to the best plan for waylaying people on the high roads, and also how to kill them without any noise in case of resistance.

prepare or suborn testimony skillfully enough to convince the jury that some dog, rabbit, elephant, monkey, etc., has been killed, in such a way as to produce blood stains which are likely to be confounded with those of the murdered victim. That I was induced to avoid specifically stating this failure of our science by no unfounded apprehension of evil results, is *proved* by the fact that after my evidence was delivered in the Larribee trial at Franklin, Pa., the prisoner's counsel, a "shrewd-witted lawyer," in order to account for spots on the defendant's boots, brought two women into court who testified that the boots were sprinkled as they stood in a corner of the kitchen, by a puppy which jumped away from them just as they got one ear cut, and ran round the room shaking its bleeding head. Further to substantiate this tale, a dog with one ear clipped was shown to the jury, and sworn to as the very one from which the blood was shed. Fortunately, however, it so happened that I had examined two spots on the prisoner's pantaloons, finding them to be human blood, in contradistinction to pheasant's blood, as he first explained them to be, and since the contrivers of this dog story apparently forgot that the pantaloons were not standing up in the boots, to be sprinkled with them, their ingenious theory failed to gain credence with the jurors, who brought in a verdict of guilty. I venture, however, to predict that from this explanatory note, *and* the essay which made it necessary, will spring a host of bloody dog tales to account for suspicious stains on the clothing, etc., of murderers, until even attorneys for the defense become themselves ashamed to put forward this thin, worn-out plea.

In regard to the supposed greater accuracy of Carl Schmidt's observation, that dried and remoistened blood corpuscles shrink nearly one-half, I desire to add that I think it is chiefly accurate concerning specimens of *crenated* corpuscles, such as form when considerable quantities of blood undergo desiccation, and will be pleased at any time to demonstrate the general correctness of my measurements of the disks in the thin films of true *blood stains* (as emphatically distinguished from *masses* of dried blood).—*Am. Jour. Med. Sci.*, April, 1875.

